# Plates over Fangs

**Published:** 1861-04

**Authors:** W. H. Atkinson


### PLATES OVER FANGS.

BY W. H. ATKINSON.

THE great sin of surgeons, especially young surgeons, is, indiscriminate slaughter of diseased or wounded parts, because they can more readily cut and remove them out of sight than, by extraordinary skill and patience, preserve them for use.

Dentists being in the aggregate more, much more ignorant of the mutual relations subsisting between parts, often imitate the more reckless surgeons, in removing organs they have neither the knowledge nor courage to treat thoroughly and restore to usefulness, and so, with a "coup de main," forever put it out of the power of even superior skill and patience to put *them* to lasting shame by saving these; which could readily be done, had it not been for their reckless display of d.xterity in mutilating.

The advantage, not to say necessity for the presence of fangs, to sustain, by lateral support, the alveolar arch, has not been, and is not at this present, properly considered by but the very few. Authority, right or wrong, overpowers all the youthful portion of timid or honest operators, and thus, for the most part, cuts off even the necessary examination of the grounds upon which it is based, thus precluding them from exercising their own unbiassed judgment, by this proscribing beforehand what is doable, and what they deem undoable, because they had not the moral courage or earnestness, in doing the very best possible for *each one;* to fully examine each case separately, irrespective of what might have been said and done by our predecessors.

The next most powerful obstruction in the way, after false authority, is the persistent determination that manifests itself in ninety per cent. of those operating, to do it in the *easiest*, not *best* manner they can contrive.

Occlusion of the dentures has been but little studied or understood; hence, successful adaptation of these, in perfect articulation, has been more a matter of accident than result of regularly taken steps towards the accomplishment of the object in view. Had this attracted the sharp attention of the sharp dentists, greater advancement in understanding the distribution of masticating forces would have been attained.

All force, acting upon receptive or passive bodies, is distributed in radiating lines from the points of contact where it is propagated, and hence, are best displayed in the contact of solids first, semi-solids next, and then the fleshy and elastic soft tissues. So that the concussions of even the most vigorous masticating, when sound teeth are well and truly set *in* the jaws, are scarcely felt enough to call attention, in communating the hardest foods, while it becomes labor to thoroughly masticate the softer and more tender foods, with the best artificial dentures, set *upon* the gums, after the natural fangs, the normal distributors of this force, have been removed, for the sake of making the so called "perfection of artificial dentures."

Nature's own perfect work is always to be chosen first, and the next choice would be the nearest approximation to this.

The question as to what comes nearest up to this standard will the more fully appear upon the statement in some detail of what constitutes perfection of the natural dentures.

Size, strength, adjustment, are the prime considerations. Size—to just fill the arch, without crowding, or leaving spaces between, into which food can wedge. Strength—to withstand the necessary force. And adjustment—to secure the most perfect adaptation of the articular faces of the various teeth, to fulfill, in the incisors, cutting; in canines, holding and tearing; and in bicuspids and molars, of finely dividing and grinding, to favor perfect insalivation. Next is form and regularity of the arrangement of the teeth, to form the terminus of the vocal tube, simulating an abruptly terminated, flattened cone, with its repressed apex gently and regularly curved downward, and finished at the edges of the incisive teeth, producing the most admirable facilities for finishing, to completeness, the modifications of voice, denominated articulation, setting free the distinct sounds we interpret into sense by common consent.

In cases where the alveolar processes are absorbed, or badly diseased, it will be necessary to extract fangs, or teeth, to insure the best results. But in all cases, where the processes are perfect and strong, up to the margin, and contain healthy fangs, the latter had better be cut down to a proper level (and have their canals obliterated by careful and nice filling), upon which to set the artificial substitute on a sufficient base.

If deposition of cement, in the form of exostosis, should project these, in a degree, from their sockets, all that is required is to trim them to the proper level, to secure the fit of the base, and the requisite stimulus to healthy action in the periosteal connections.

As long as the fangs remain in a healthy condition, the insertion of muscle is not changed; but as soon as the fangs are removed, and complete absorption is effected, the points of insertion become in some degree changed, the lips thinned and contracted, simulating great age, even in young persons. *Perfect* restoration of this condition is out of our power, by any known means. I say this, in full view of the history of "plumpers" and projecting methods, so successfully resorted to, to restore the fullness of face lost in every case where indiscriminate extraction has prevailed. The first case of *perfect* restoration has yet to be presented. Pause, then, before you decide to mutilate!

If you have not the ability to decide satisfactorily upon saving or extracting, for your own after peace and the honor of the profession, take advice from the competent, or dismiss the applicant, with the injunction to seek further light, and obey the dictates of reason and humanity.

Among dentists, the neglecting to remove fangs that ought to be promptly extracted, is not so frequent as the removing of fangs and whole teeth that ought *not* to be taken out. Where *one* commits the first sin, *ten* unblushingly exult in and boast of doing the latter, in their ignorance, supposing that they set themselves at the head of the profession, by the exercise of their "thorough skill" in "clearing the mouth" for a beautiful (?) set of artificial SUBSTITUTES.

Teeth that have been without antagonists for a number of years, in otherwise healthy mouths, will be apt to be exostosed, or to have their fangs shortened by absorption, to a considerable extent. The best means of diagnosis for such cases, doubtless, is great familiarity with them; but some of the more pathognomic signs are, elevation above the normal plane of the grinding faces of the teeth yet enjoying the presence of their opposites; and a certain kind of "looseness," or mobility, devoid of evidence of periostitis. I never have seen both these symptoms present *with entire* alveolar processes, unless exostosis and absorption, one or both, were present. With few exceptions, such teeth had better be removed. Study *hard* and *long;* religiously *diagnose every* case; then follow your first "*instinctive feeling*," and, in the majority of cases, you are SAFE.

CLEVELAND, Dec. 14, 1860.

